# Neuronal overexpression of hTDP-43 in *Caenorhabditis elegans* impairs motor function

**DOI:** 10.17912/micropub.biology.000768

**Published:** 2023-04-19

**Authors:** Mandy Koopman, Lale Güngördü, Renée I. Seinstra, Ellen A.A. Nollen

**Affiliations:** 1 European Research Institute for the Biology of Ageing, University of Groningen, University Medical Centre Groningen, The Netherlands

## Abstract

Transactive response DNA binding-protein 43 (TDP-43) is a conserved RNA/DNA-binding protein with a role in RNA metabolism and homeostasis. Aberrant TDP-43 functioning has been considered a major culprit in amyotrophic lateral sclerosis (ALS). *Caenorhabditis elegans* can be used to phenocopy ALS *in vivo* . Since disrupted locomotion is a strong readout of toxicity, we examined multiple motor phenotypes of a *C. elegans* model expressing human wild-type *TDP-43* ( *hTDP-43* ) pan-neuronally. Our data reveal that impaired locomotion includes more than the common deficits in crawling capacity and the presence of early-onset paralysis. We show that reduced thrashing, abnormal coiling, and decreased pharyngeal pumping are also observed, in a temperature-dependent fashion.

**Figure 1. Pan-neuronal expression of hTDP-43 in C. elegans causes widespread movement abnormalities:**
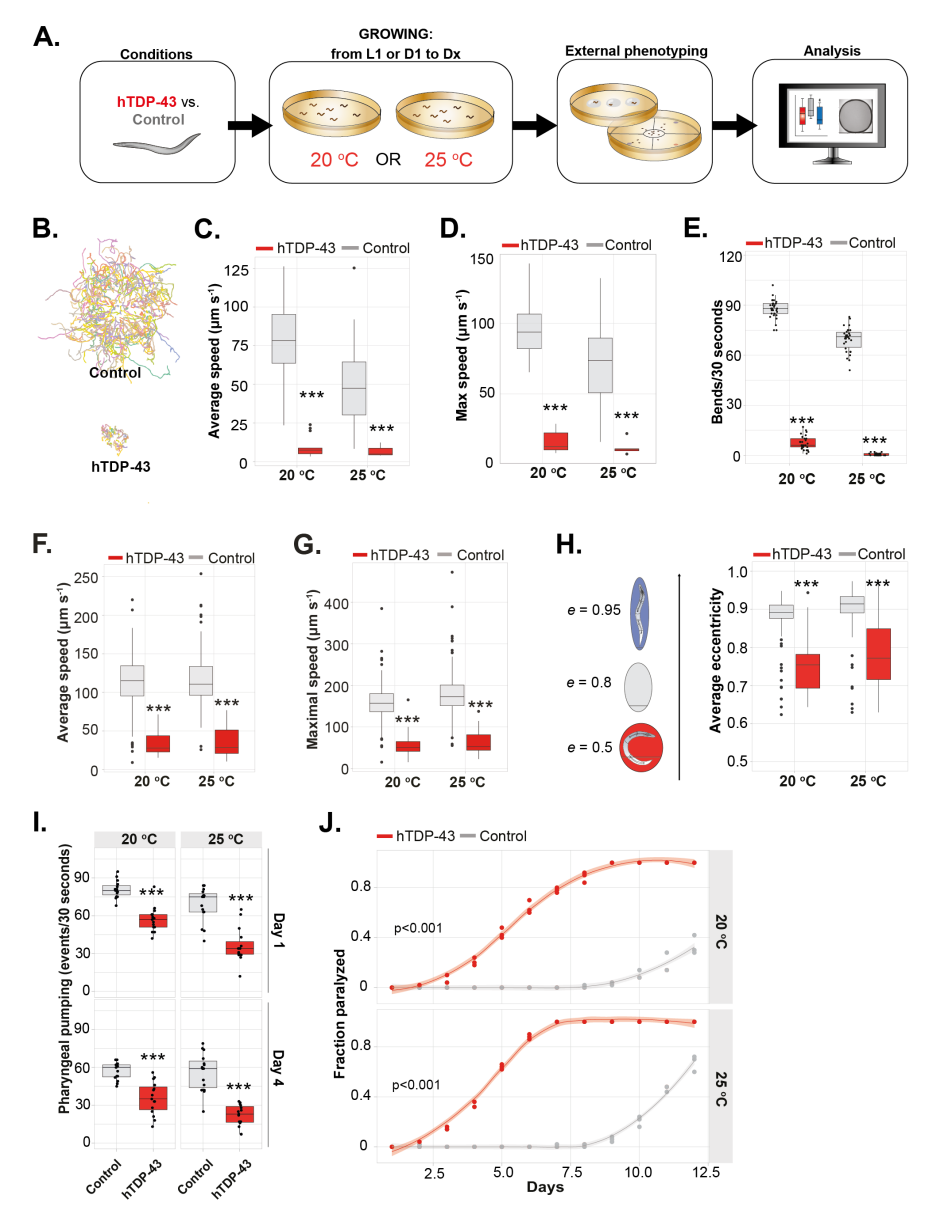
**A)** Schematic showing the experimental outline for the external phenotyping. For all graphs the following color-coding is used: hTDP-43: red, control: grey. Worms were grown from L1 at 20 °C or 25 °C and tested at adulthood D1 unless stated otherwise. **B)** A representative crawling-map of 10 minutes generated by the WF-NTP software (20 °C). **C)** The average crawling speed (μm s ^-1^ ) of hTDP-43 and control worms, *n* = 10-65, Mann-Whitney U test 20 °C: p<0.001, 25 °C: p<0.001. One representative experiment of a triplicate is shown. **D)** The maximum crawling speed (μm s ^-1^ ; 90 ^th^ percentile) of hTDP-43 and control worms, *n* = 10-65, Mann-Whitney U test 20 °C: p<0.001, 25 °C: p<0.001. One representative experiment of a triplicate is shown. **E)** Bends per 30 seconds in liquid, counted manually. *n* = 15, Mann-Whitney U test 20 °C: p<0.001, 25 °C: p<0.001. One representative experiment of a triplicate is shown. **F)** The average movement speed (μm s ^-1^ ) during thrashing of hTDP-43 and control worms, *n* = 50-350, Mann-Whitney U test 20 °C: p<0.001, 25 °C: p<0.001. One representative experiment of a triplicate is shown. **G)** The maximal movement speed (µm s ^-1^ ) during thrashing of hTDP-43 and control worms, *n* = 50-350, Mann-Whitney U test 20 °C: p<0.001, 25 °C: p<0.001. One representative experiment of a triplicate is shown. **H)** The eccentricity of hTDP-43 (used as a measure of how circular an ellipse is) and control worms in liquid, *n =* 50-400, Mann-Whitney U test 20 °C: p<0.001, 25 °C: p<0.001. One representative experiment of a triplicate is shown. **I)** Pharyngeal pumping expressed in events per 30 seconds, *n =15* , two-way ANOVA at both 20 °C and 25 °C (age, genotype: p<0.001, interaction: n.s.) with post-hoc Sidak’s. One representative experiment of a triplicate is shown. **J)** Paralysis-assay of hTDP-43 and control worms (50 worms per replicate), *n = 3* , Log-rank Mantel-Cox test 20 °C: p<0.001, 25 °C: p<0.001. Worms were grown from L1 at 20 °C and subsequently transferred at D1 to 20 °C or 25 °C. Error bars represent the S.E.M. and transparent areas in line-graphs represent 95% confidence interval. For boxplots: separate dots in C, D, F, G, H represent outliers outside the [Q1 – (1.5) x IQR (interquartile range), Q3 + (1.5) x IQR] range. Separate dots in E and I represent the individual data points. *: p ≤ 0.05, **: p ≤ 0.01, ***: p ≤ 0.001.

## Description

After observing TDP-43 pathology in a *Caenorhabditis elegans* model expressing human *TDP-43* pan-neuronally (hTDP-43 worms) (Koopman et al., 2023a) we investigated the functional consequences of these molecular features by assessing global motor function ( **Figure 2A** ). Movement phenotypes like crawling and thrashing have proven to be auxiliary in studies concerning the pathology of muscles and neurons (Brignull et al., 2006; Sorrentino et al., 2006; Ash et al., 2010; van Ham et al., 2010; Hahm et al., 2015) . Due to differences in kinematics, the pattern of muscle activity and involvement of sensory neurons, trashing (i.e. the frequency of lateral bends in liquid, c-shapes) and crawling (e.g. the two-dimensional sinusoidal wave-like movement, s-shapes) provide distinct but complementary information about the (neuromuscular) health of *C. elegans* (Karbowski et al., 2006; Korta et al., 2007; Pierce-Shimomura et al., 2008; Hahm et al., 2015; Fang-Yen et al., 2015) *.* In addition, rhythmic movements that are dependent on interactions between the nervous system and muscles can also be studied by assessing the pharyngeal pumping rate (Trojanowski et al., 2016) . Pharyngeal pumping rates decrease with age and provide another fitness parameter for *C. elegans* (Chow et al., 2006) .

Using automated tracking we found that the crawling capacity of hTDP-43 animals, expressed as average and maximal translocation speed, was impeded ( **Figure 2B-D** ). The slow movement of hTDP-43 worms was accompanied by a distinctive ‘uncoordinated’ phenotype as previously described (Ash et al., 2010) . Similarly, thrashing frequency and consequential translocation were reduced by the presence of hTDP-43 ( **Figure 2E-G** ). Here, impaired ‘thrashing’ coincided with an ‘coiler’ phenotype ( **Figure 2H** ), a posture that is often observed in mutants with abnormal cholinergic signaling (Rand et al., 1984; Sluder et al., 2012) . Given the large effects of TDP-43 on locomotion, the decrease in pharyngeal pumping appeared relatively moderate ( **Figure 2I** ). Impaired locomotion early in life progressed to complete paralysis at adulthood day 9 (20 °C) or day 6 (25 °C) ( **Figure 2J** ). Noteworthy, the mechanical stimuli provided to assess paralysis revealed a clear ‘shrinker’ phenotype characteristic for GABAergic dysfunction (Schuske et al., 2004) . Together, a higher environmental temperature consistently enhanced most of the phenotypic abnormalities of hTDP-43 worms. Importantly, similar temperature-dependent effects, but to a lesser extent than those seen in hTDP-43 worms, were observed in control worms ( **Figure 2C-I** ).

In conclusion, our data shows that neuronal expression of TDP-43 impairs locomotor function in *C. elegans* . Moreover, the existence of more specific features such as coiling, touch-induced shrinking and uncoordinated movement provide insights into the neuronal systems affected by TDP-43.

## Methods

**Strains and maintenance**

Standard conditions were used for *C. elegans* propagation at 20 °C (Brenner, 1974) . Animals were age-synchronized by hypochlorite bleaching and subsequently allowed to hatch overnight in M9 buffer at 20 °C. For experiments age synchronized L1s were cultured for 72h at either 20 °C or 25 °C on NGM plates seeded with OP50 before being tested, unless stated differently.

**Thrashing and crawling**

For crawling ability, we recorded the free crawling of worms on empty NGM agar plates for 30 seconds at 20 fps (except for the generation of crawling maps, for which worms were recorded for 10 min. at 3 fps) with the WF-NTP platform. Determination of crawling speed and generation of crawling maps was performed using the WF-NTP software (Koopman et al., 2020) . Data on thrashing were acquired by transferring worms to empty NGM agar plates flooded with M9 and subsequently recording them for 30 seconds at 20 fps. The WF-NTP software was used to derive the thrashing frequency, but also changes in eccentricity. Since uncoordinated worms do skew centroid-based trackers, we additionally recounted (at least) one experiment manually by counting at least 15 randomly chosen worms per video.

**Paralysis**

Synchronized worms were cultured on standard NGM plates seeded with OP50 at 20 °C. At day 1 of adulthood, 50 worms per condition (unless stated differently) were transferred to 6-cm NGM plates containing FUdR (10 worms per plate). Plates were kept at 20 °C or 25 °C, as described in the figures. Animals were tested for paralysis every day by tapping their nose/tail with a platinum wire as described previously (Cohen et al., 2006) . Worms that failed to show a touch-response (i.e., moving their nose, but not their body) were scored as paralyzed. Worms that did not move, did not show a touch-response, and had no pharyngeal pumping were considered dead and were excluded from analysis.

**Pharyngeal pumping**

Worms were freely moving on freshly seeded NGM plates and were examined under an optical microscope to determine pumping rates by visual observations. One pharyngeal pump was defined as a complete forward and backward movement of the grinder in the pharynx (Hobson et al., 2006) . The rate was counted for 30 seconds.

## Reagents

**Table 1:** Strains used

**Table:** 

**Strain**	**Description**	**Genotype**	**Remark**
N2	Bristol wild isolate	Wildtype	
OW1601	CL6049 6x backcrossed with N2 . Named: hTDP-43 worms.	*dvIs62* [ *snb-1p::hTDP-43/3’long UTR + mtl-2p::GFP* ]X	Provided by Chris Link
OW1603	CL2122 6x backcrossed with N2 . Named: control worms.	*dvIs15* [( *pPD30.38) unc-54 (vector) + (pCL26)mtl-2p::GFP* ]	Provided by Chris Link
